# Effect of Spousal Loss on Depression in Older Adults: Impacts of Time Passing, Living Arrangement, and Spouse’s Health Status before Death

**DOI:** 10.3390/ijerph182413032

**Published:** 2021-12-10

**Authors:** Yu-Chan Hung, Yong-Hsin Chen, Meng-Chih Lee, Chih-Jung Yeh

**Affiliations:** 1 Department of Public Health, Chung Shan Medical University, Taichung 40201, Taiwan; ych0610@gmail.com (Y.-C.H.); a6328539@gmail.com (Y.-H.C.); 2 Department of Occupational Safety and Health, Chung Shan Medical University Hospital, Taichung 40201, Taiwan; 3 Institute of Medicine, Chung Shan Medical University, Taichung 40201, Taiwan; 4 Department of Family Medicine, Taichung Hospital, Ministry of Health and Welfare, Taichung 40201, Taiwan; 5 Institute of Population Health Sciences, National Health Research Institutes, Miaoli 35053, Taiwan

**Keywords:** spousal death, depression, older adults

## Abstract

In addition to increasing the mortality among older adults, spousal death (SD) increases their risk of depression. This study explored the factors affecting depression among widowed older adults to provide health care strategies for successful aging. A total of 710 adults older than 60 years completed a questionnaire before and after their spouses’ deaths. The survey data included age, sex, ethnic group, education level, financial station socioeconomic status, SD (including time point), smoking status, alcohol consumption, self-rated health status, Center for Epidemiologic Studies Depression Scale score, mobility, and degree of support from relatives and friends. The proportion of participants with depression after SD was 1.7 times that of before SD (*p* < 0.0001). Worsened mobility (odds ratio [OR] = 1.3, *p* < 0.01), low self-rated health status (OR = 0.5, *p* < 0.01), and a high degree of support from relatives and friends (OR = 1.5, *p* < 0.01) had a significant positive correlation with depression after SD. The proportion of depression that occurred within 6 months after SD was 6.0 times higher than that of depression before SD. Participants who lived alone after losing their spouses who were healthy before their deaths exhibited a significantly increased proportion of depression after their spouses’ deaths. Male sex, spouse’s health, and the period of 6 months after SD are risk factors for depression in older adults. The maintenance of mobility, positive self-rated health status, and a shorter period of depression after a spouse’s death result in more favorable adaptability among women. Social workers or family members should focus on older adults whose spouses died unexpectedly or within the last 6 months. Living with family members after SD can alleviate depression in older adults.

## 1. Introduction

Spousal death (SD) has a wide range of health effects on older adults like mental, social, behavioral, and biological issues [1]. It is closely related to the risk of death in elderly [2] and increases the mortality of various diseases [3]. In particular, the mortality of old people increases in the first few months after a spouse’s death [4,5,6]. The lifestyle changes caused by SD increase the incidence of almost all types of cancer [7]. It is also a risk factor for stroke [8] and is related to the severity of cardiovascular disease [9]; moreover, widowed men have an increased risk of type II diabetes [10]. In 1980, a study demonstrated that in addition to medical technology and drugs, a decrease in the proportion of SD was a reason for the decline in disabilities among older adults in the United States [11].

Although the emotional response of older adults after their spouses’ deaths may gradually decrease over time [12], their period of grief is several years long rather than temporary [1,13,14,15], and the sadness caused by a spouse’s death affects the occurrence of depression [16]. Similar to sorrow, depressive symptoms continue to occur in elderly at a relatively high level [17,18].

The psychological, social, behavioral, and physical conditions of the widowed are worse than those who are currently married or cohabiting. This includes depression, decreased social connections, unhealthy lifestyles, and poor cardiovascular health [1]. Another study also indicated that spouse loss increases the degree of depression and that it will last for several years [14].

Depression is a common mental illness among older adults [19] and a population-based cohort study also showed that age-standardized depression prevalence in older adult was 6.8% [20]. Old people with depression have a relatively high rate of disease comorbidity as well as increased mortality and risk of disability and suicide [21,22,23]. Older adults living independently from family or friends are particularly susceptible to depression [24]; however, social isolation is more likely to cause depression in this population compared with people who live alone [25]. Although family support can reduce the difference in depressive symptoms between old people who experienced SD and other older adults [26], the absence of children does not increase loneliness or depression in widowed elderly [27]. In addition, help from children and the sharing of living arrangements with them yield reduced depression symptoms and favorable self-rated health [28]. This study explored the major effects of SD on older adults’ depression and whether, in addition to SD, the changes in lifestyle habits, interpersonal relationships, or living environments after a spouse’s death are influencing factors. The results may provide health care strategies for the successful aging of older adults.

## 2. Materials and Methods

### 2.1. Study Design and Sampling

The TLSA (Taiwan Longitudinal Study of Aging) data source of this study is the Institute of Family Planning, which was the predecessor of the Health Promotion Bureau of the Ministry of Health and Welfare, Taiwan. Stratified three-stage random sampling was adopted to select 4412 individuals from the registered population of Taiwan who were older than 60 years at the end of 1988. Of the selected individuals, 710 completed the questionnaire before and after their spouses’ deaths. A questionnaire survey was conducted by special investigators through interviews to collect baseline data. For selected participants who could not respond to the questionnaire because of conditions such as unconsciousness, severe illness, or deaf-mutism, family members or caregivers who understood their situations could answer for them.

### 2.2. Questionnaire

A total of six longitudinal surveys were conducted in 1989, 1993, 1996, 1999, 2003, and 2007. Collected data included age, sex, ethnic group, education level, financial status, smoking status, alcohol consumption, and self-rated heath status. The simple version of the 10-item Center of Epidemiological Studies Depression Scale (CESD) was used to measure depression, and a score of ≥10 indicated depressive symptoms. In the mobility investigation, participants were asked if they had difficulty squatting, raising both hands, picking up objects with their fingers, lifting an 11-kg object, walking up two to three floors, or walking 200–300 m, with higher scores indicating poorer mobility. In the survey on relative and friend (RF) support, participants were asked if they believed that their RF were willing to listen to their worries, whether their RF cared about them, whether they were satisfied with the level of care expressed by their RF, and whether their RF were hypercritical to what they did. In addition, the participants were asked whether their RF would ask for their opinions when making decisions or during discussions. In terms of the quantification of support from RF, a higher score indicated lower support. Regarding the questionnaire items, reduced-rank regression was first adopted to obtain the factor loadings for SD, after which the scores for mobility and RF support were acquired. Therefore, mobility and RF support scores were used to represent mobility and RF support in the later section of this study. The dimensions measured in the questionnaire are shown in Table 1.

### 2.3. Statistical Methods

We conducted reduced rank regression to analyze the factor loading of mobility items and social support items. Higher RRR score in mobility means worse mobility function and higher RRR score in social support means higher level of social support.

The McNemar’s Chi-square test was conducted to determine whether each participant presented significant differences before and after SD, and the effect of variable adjustment on SD was analyzed using multiple logistic regression. SAS 9.4 (SAS Institute Inc., Cary, NC, USA) was employed with a significance level of 0.05.

## 3. Results

### 3.1. Factor Loadings of Mobility and RF Support

Table 2 presents the calculation results for the factor loadings of mobility and RF support.

The factor loading for each mobility was positive and greater than 0.2, indicating a positive correlation between the degree of disability and depression. It can be found that the factor loading of lower limb activity items were greater than 0.43 (walking 200–300 m, climbing up 2–3 floors and squatting), while the upper limb activity items were between 0.35–0.42 (grasping with the fingers, carrying 11 kg weight and raising the arms up). This means that lower limb activity items have a higher impact on depression than upper limb activity items.

The factor loadings for the surveyed items of RF support were negative and greater than 0.2, implying a negative correlation between support level and depression. It can be found that the absolute value of factor loading of “care from significant others” was greater than 0.5, while other items were between 0.27 to 0.4 (“talk”, “ask opinion”, and “complain”). This means that “care from significant others” have a higher impact on depression than other social support items.

### 3.2. Participants’ Characteristics

Table 3 presents the participant demographic variables and the descriptive statistics for SD time point and Time since SD. Of the 710 participants, 256 were men and 454 were women. The participants were divided into six groups (on the basis of the spouses’ age at death), four education levels (from primary school to university and above), and four ethnic groups. The majority of the participants were female (63.9%), educated at the primary level (41.6%), and Fukinese (71.8%).

The years 1989, 1993, 1996, 1999, 2003, and 2007 were used as the points of tangency for the time of SD occurrence. As shown in Table 3, the highest percentage of SD was between 1999 and 2003 (27.6%). This situation also reflects that participants will experience spouse death from the time they started participating in the study to a certain age.

Time since SD was divided into five levels, namely less than 3 months, 3–6 months, 6–12 months, 12–24 months, and more than 24 months. There were a total of 42.7% of participants where time since SD was more than 24 months.

### 3.3. Pre and Post Spouse Death (SD)

Table 4 presents data before and after SD according to participant sex. No significant difference was observed in income satisfaction between men and women before and after SD. A decline in the proportions of both men (ORm = 4.38, *p* < 0.0001) and women (ORm = 7.0, *p* < 0.05) who smoked was observed. However, neither men nor women exhibited a significant difference in alcohol consumption before and after their spouses’ deaths. In terms of self-rated health, men did not exhibit a significant difference before and after SD, whereas the proportion of women who believed that they had favorable health declined after SD (ORm = 1.40, *p* < 0.05). In Table 4, diff represents the result of subtracting the post-SD score from the pre-SD score. Men exhibited a significantly decreased degree of depression after SD (mean = 1.52 ± 6.09, *p* < 0.0001), whereas the women’s degree of depression increased significantly (mean = −1.31 ± 7.49, *p* < 0.01). A mobility score diff of <0 indicates improved mobility. Men exhibited reduced mobility after SD (mean = 0.33 ± 1.25, *p* < 0.0001), whereas the mobility of women improved (mean = −0.52 ± 1.50, *p* < 0.0001). Neither men nor women exhibited a significant difference RF support scores after SD, indicating no significant change in support from relatives and friends after SD.

### 3.4. Effect of SD on Depression

Table 5 presents the multinomial logistic regression analysis of the effect of SD on depression. Model 1 consists of the simple effect without any variable adjustment; the mobility score was adjusted in Model 2, self-rated health was adjusted in Model 3, and the RF support score was adjusted in Model 4. Under the Model 1 SD effect, the proportion of depression among the participants after SD was 1.7 times that of before SD (*p* < 0.0001), whereas the SD effects of Models 2, 3, and 4 resulted in a proportion of depression after SD 1.5–1.7 times that of before SD, and the increase was significant. The odds ratios (ORs) of the adjustment variables in Models 1–4 were all significant, indicating that worsened mobility (OR = 1.3, *p* < 0.01), declined self-rated health status (OR = 0.5, *p* < 0.01), and higher RF support (OR = 1.5, *p* < 0.01) were related to a higher proportion of depression. After variable adjustment, the SD effect on men remained significant, and the ORs of the adjustment variables were nonsignificant. After the mobility score (Model 2) and favorable self-rated health status (Model 3) were adjusted, the OR of the SD effect on women became nonsignificant, whereas the ORs of the adjustment variables became statistically significant (OR = 1.3, *p* < 0.01; OR = 0.4, *p* < 0.01). Mobility, self-rated health, and RF social support are associated with depression in total subjects and in female; but in male, mobility, self-rated health, and RF social support are not significantly associated with depression. In female, the SD effect on depression disappeared after adjusting the mobility variable.

Table 6 indicates that the proportion of post-SD depression was 6.0 times, 0.9 times, 1.7 times, and 1.3 times that of pre-SD depression when Time since SD was less than 6 months (OR = 6.0, *p* < 0.0001), 6–12 months (OR = 0.9, *p* > 0.05), 12–24 months (OR = 1.7, *p* < 0.05), and ≥24 months (OR = 1.3, *p* > 0.05), respectively. The proportion of depression decreased rapidly after 6 months. In the sex-stratified analysis, only men had a significantly higher proportion of post-SD depression compared with pre-SD depression in the 12–24 months after SD (OR = 2.38, *p* < 0.05), whereas women exhibited a significantly higher proportion of post SD depression less than 6 months after SD (OR = 4.0, *p* < 0.01). The remaining variables yielded no significant changes. Regardless of gender, the depression proportion is obviously high in six months after spouse death, which declines after six months and without statistical difference for proportion after 24 months. Men suffered depression more than women in every period, and in particular, SD effect on depression, in period 12 and 24 months after SD, is statistically significant in men.

In the analysis of post-SD living arrangements, Table 6 indicates that the proportion of depression among widowers and widows living alone and those living with family members was 2.3 times (OR = 2.3, *p* < 0.01) and 1.5 times that before SD (OR = 1.5, *p* < 0.01). A further sex stratification analysis revealed that men who live alone after SD exhibited more depression (OR = 3.0, borderline significance) and men who lived with their families after SD exhibited significantly more severe depression compared with before SD (OR = 2.3, *p* < 0.01). In the stratified analysis of SD effect on depression in different living arrangements, the results for female (OR = 2.0; OR = 1.3) are similar to the trends for male, but there is no statistically significant association under “living alone” or “living with family members”.

In terms of the stratified analysis of spouse’s health status, Table 6 indicates that a significantly large proportion of participants whose spouses had a favorable health status exhibited post-SD depression (all participants; OR = 2.0, *p* < 0.01, men: OR = 2.3, *p* < 0.05, women: OR = 1.8, *p* < 0.05). However, no significant difference was observed in the proportion of widowers and widows with post-SD depression whose spouses were in poor health.

## 4. Discussion

Our findings indicate that male sex, spouse’s health, and the period of 6 months after SD are risk factors for depression in elderly. The maintenance of mobility, positive self-rated health status, and a shorter period of depression after a spouse’s death result in more favorable adaptability among women. Living with family members after SD can alleviate depression in older adults.

The strength of our study: (1) as a longitudinal study, this study can provide clearer causal relationship between SD and depression, (2) TLSA is a national survey that has a representative sample of old people in Taiwan, (3) this study used self-matched to explore the effect of SD on depression in older adults.

Smoking among men and women declined after SD, whereas alcohol consumption among the participants exhibited no significant difference, which was inconsistent with the finding of increased tobacco and alcohol abuse after SD in previous studies [29,30,31,32]. The proportion of self-rated health decline after SD increased significantly among women. This was consistent with the findings of previous studies indicating that widowed elderly have poorer self-rated health [33,34] and that SD negatively affects health [35]. Men exhibited an obvious decline in mobility after SD, whereas the mobility of women improved, indicating a sex difference. Previous studies have demonstrated that women’s mobility after SD is inferior to that of women whose spouses are still alive [36,37]; however, another study revealed that widows in Taiwan are more active in recreational activities compared with married women [38]. The disparity between the present research results and the literature may be attributable to cultural differences. Although RF support did not differ significantly after SD, depression and the degree of support from relatives and friends were positively correlated. This may be explained by the increased care that participants received from older adults due to their depression. However, RF support had little influence on SD effects after variable adjustment, indicating that such support cannot significantly alleviate depression. According to attachment theory, support from friends cannot compensate for the loss of someone to whom an individual had an attachment [39], and attachment problems are particularly prevalent among old people [40]. These studies may explain why support from relatives and friends cannot effectively alleviate depression.

Men exhibited a decline in depression after SD whereas women presented increased depression. However, after adjusting for mobility, self-health rating, and RF support, the proportion of men who had depression after SD was greater than that of women, indicating that SD had a significant effect on depression among men (more than 2.3 times that before SD). In other words, adopting approaches to alleviate the effects of SD on depression is more difficult for men. The proportion of women with depression after SD increased significantly (1.5 times); however, after adjusting for mobility and self-rated health, the original effect of SD on depression was explained by the adjustment variables. In addition, the adjustment variables were significantly correlated with depression. In other words, the effects of SD on depression could be alleviated or reduced in women through more favorable mobility and a favorable perception of their own health, a phenomenon not observed in men. This result is consistent with the findings of previous studies, which demonstrated that depressive symptoms caused by SD have a stronger effect on men, whereas women have superior adaptability [41,42,43]. Moreover, recreational physical activity has a preventive effect on depression in women [44]. Increased participation in recreational activities is beneficial for widows, and changes in leisure activities after SD have a greater effect on health and disability compared with aging [45]. These findings corroborate the aforementioned research results.

The proportion of depression was obvious within 6 months after SD; however, this trend gradually slowed after 6 months. For women, this increase was not significantly different after 6 months (however, a significant difference was observed among men between 12 and 24 months after SD). After 24 months, both men and women exhibited no significant difference in depression before and after SD; therefore, the effect of SD can be alleviated over time. A previous study demonstrated that older adults experience relatively high levels of depressive symptoms within 2 years after their spouses’ deaths [17]. The proportion of depression among women remained high within 6 months after SD; however, this proportion decreased to pre-SD levels after 1 year [18]. These conclusions are consistent with our research results. This study indicated that SD has a shorter effect on depression in women compared with men. Past research has also demonstrated that the amount of time after a spouse’s death affects morale and social engagement [46]. The relatively short duration of depression among women after SD may cause them to have a more positive self-rated health status or participate in social engagement, which may also explain why women have more favorable adaptability to depression caused by SD [41,42].

The proportion of depression increased after SD regardless of the participants’ living arrangements; however, depression in old people living alone after their spouses’ deaths exhibited a greater increase than those who live with family members. Although the aforementioned trend persisted after sex stratification, depression among only men who lived with their families exhibited statistical significance. Therefore, both living alone and living with family members significantly affects depression. However, previous research has demonstrated that living alone is not correlated with depression [25]. After adjustment for demographic variables, health status, social support, and financial status, the effect of living alone on depression disappeared [47]. Although the same trend was observed in this study, participants who lived alone still exhibited a relatively high proportion of depression. Older adults whose spouses were healthy exhibited significantly higher proportion of depression after SD, a trend that was clearly observed in both men and women. However, older adults whose spouses had poor health exhibited no significant change in the proportion of depression after SD. Past studies have demonstrated that elderly who have lost their spouses are more likely to be depressed if they were not the primary caregiver of their sick spouses [48], and the accidental death of a spouse may increase the risk of depression [49]. The aforementioned results can be used to illustrate the phenomena revealed in this study.

By conducting a long-term investigation of widowed elderly, this study demonstrated that SD is a risk factor for depression among older adults in Taiwan, and men and women adapt differently to their spouses’ deaths. Women’s shorter duration of depression after SD, their ability to maintain their mobility, and their more favorable self-rated health result in an increased willingness to participate in activities. This enables them to have superior adaptability to the effects of SD. Older adults are at risk of depression within 6 months after SD. Relatives, friends, and social workers should increase their attention to and care of them during this period. In terms of living arrangements, encouraging elderly to live with family members or friends after SD can reduce the incidence of depression.

Important scientific contributions and implications of this study are summarized. Firstly, six months after spouse death is a high-risk period for depression. Families, friends, relatives, or social workers, have to pay more attention to widowhood elderly in six months. Secondly, older men more vulnerable to depression than women after spouse loss. In male, life assistance, instrumental and emotional supports are important practical issues after spouse loss. Third, those who live alone after spouse death suffered more depression than those who live with their family members. The living arrangement is very important to elderly, which affects the rate of adjustment after spouse loss. Fourth, the spouse was in good health before death, which means the great psychological impact of this “unexpected” event. Frequent contact, more care and support are needed for widowhood facing this unexpected event.

There are some limitations though we explore several important issues for spouse loss and depression. Firstly, marital satisfaction, marital relationship, marital conflict, and the length of time they were married, were not collected in this study. Lacking these marital factors might limit us to elucidate their important roles in the rate of grief and grief adaptation. Secondly, grief and major depressive disorder share similar symptoms, such as intense sadness, insomnia, poor appetite, weight loss, etc. This study uses the CESD-10 depression scale, without the grief scale measurement, so it is difficult to distinguish grief and depression. Further studies may compare the differences of depression scale and grief scale in widowhood, or perform single-item analysis of “intense sadness”, “insomnia”, “poor appetite”, or “weight loss” in widowhood, to distinguish grief and depression.

According to our findings, if the spouse was in good health before death, his/her death (“unexpected” event) will make the great psychological impact to the other. People around these older adults should pay more attention to them. We also found that living with families after SD can alleviate depression in elderly.

We suggested that these older adults who have experienced unexpected widowhood can take turns to live with their children, or travel with good friends, and receive care from families and friends.

## 5. Conclusions

Our findings indicate that male sex, spouse’s health, and the period of 6 months after SD are risk factors for depression in older adults. The maintenance of mobility, positive self-rated health status, and a shorter period of depression after a spouse’s death result in more favorable adaptability among women. Social workers or family members should focus on elderly people whose spouses died unexpectedly or within the last 6 months. Living with family members after SD can alleviate depression in elderly.

## Figures and Tables

**Table 1:** Dimensions measured in the questionnaire.

Demographic factors	
	Age
	Sex
	Ethnic group
	Education level
**Healthy lifestyle**	
	Smoking status
	Alcohol consumption
	Self-rated heath status
**Depression measurement**	
	Center of Epidemiological Studies Depression Scale (CESD)
**Mobility investigation (Have difficulty with the following actions)**	
	Squat
	Raise both hands
	Pick up objects with fingers
	Lift an 11-kg object
	Walk up two to three floors or 200–300 m
**Relative and friend (RF) support**	
	The RF were willing to listen to their worries
	The r RF cared about them
	Satisfaction with caring from RF
	The RF were hypercritical to what they did

**Table 2:** Factor loading derived by reduced rank regression: using pre-SD mobility and social support items.

Mobility Items	Factor Loading	Social Support Items	Factor Loading
Walking 200–300 m	0.44	Satisfaction with care from significant others	−0.57
Climbing up 2–3 floors	0.43	Willingness of significant others to care for you	−0.54
Squatting	0.43	Willingness of significant others to talk with you	−0.40
Grasping with the fingers	0.42	Willingness of significant others to ask your opinion	−0.38
Carrying 11 kg weight	0.37	Significant others’ complaints to you	−0.27
Raising the arms up	0.35	--	

SD: Spouse Death; Factor loadings with absolute value ≥0.2 are shown in bold; higher RRR score in mobility means worse mobility function; higher RRR score in social support means higher level of social support.

**Table 3:** Characteristics of study subjects.

	Total Subjects (*n* = 710)		Male (*n* = 256)		Female (*n* = 454)	
	*n*	%	*n*	%	*n*	%
Age of SD						
60–	39	5.5%	5	2.0%	34	7.5%
60–65	87	12.3%	20	7.8%	67	14.8%
65–70	148	20.9%	49	19.1%	99	21.8%
70–75	160	22.4%	54	21.1%	105	23.0%
75–80	165	23.2%	68	26.6%	97	21.4%
80+	112	15.8%	60	23.4%	52	11.5%
Education						
illiterate	320	45.1%	52	20.3%	268	59.0%
Elementary	295	41.6%	146	57.0%	149	32.8%
Junior/senior high	85	12.0%	50	19.5%	35	7.7%
Above college	10	1.4%	8	3.1%	2	0.4%
Ethnicity						
Fukinese	510	71.8%	172	67.2%	338	74.5%
Hakka	113	15.9%	46	18.0%	67	14.8%
Mainlander	75	10.6%	38	14.8%	37	8.2%
Other	12	1.7%	0	0.0%	12	2.6%
SD between						
1989–1993	120	16.9%	43	16.8%	77	17.0%
1993–1996	110	15.5%	54	21.1%	56	12.3%
1996–1999	138	19.4%	38	14.8%	100	22.0%
1999–2003	196	27.6%	70	27.3%	126	27.8%
2003–2007	146	20.6%	51	19.9%	95	20.9%
Time since SD						
0–3 months	40	5.6%	16	6.3%	24	5.3%
3–6 months	47	6.6%	18	7.0%	29	6.4%
6–12 months	105	14.8%	37	14.5%	68	15.0%
12–24 months	215	30.3%	83	32.4%	132	29.1%
>24 months	303	42.7%	102	39.8%	201	44.3%

**Table 4:** Characteristics of study subjects: pre and post spouse death (SD).

Categorical Variables				Male Post SD		ORm			Female Post SD		ORm
				+	−				+	−	
Satisfied Income	+: Yes −: No	Pre SD	+	69	45	0.80 (NS)	Pre SD	+	104	68	0.94 (NS)
			−	56	86			-	72	210	
Smoking habit	+: Yes −: No	Pre SD	+	98	35	4.38 (***)	Pre SD	+	15	7	7.0 (*)
			−	8	115			−	1	431	
Alcohol drinking	+: Yes −: No	Pre SD	+	58	31	1.11 (NS)	Pre SD	+	18	20	1.11 (NS)
			−	28	139			−	18	398	
self-rated health	+: Yes −: No	Pre SD	+	162	44	1.42 (NS)	Pre SD	+	207	85	1.40 (*)
			−	31	19			−	61	101	
		Mean		(sd)		*p*	Mean		(sd)		*p*
Depressive: Increasing means more depressive	diff	1.52		(6.09)		***	−1.31		(7.49)		**
Mobility: Increasing means even worse	diff	0.33		(1.25)		***	−0.52		(1.50)		***
RF support: Increasing means more support	diff	0.11		(1.16)		NS	0.05		(1.15)		NS

* *p* < 0.05, ** *p* < 0.01, *** *p* < 0.0001, RF support: relative and friend support.

**Table 5:** Effect of spouse death on depression: multiple conditional logistic regression.

	Total		Male		Female	
	OR	*p*	OR	*p*	OR	*p*
Model 1:						
SD Effect (Post SD vs. Pre SD)	1.7	***	2.6	**	1.5	*
Model 2:						
SD Effect(Post SD vs. Pre SD)	1.5	**	2.3	**	1.2	NS
Mobility (higher score means worse)	1.3	**	1.3	NS	1.3	**
Model 3:						
SD Effect(Post SD vs. Pre SD)	1.7	**	2.6	**	1.4	#
Self-rated health (good vs. not good)	0.5	**	0.8	NS	0.4	**
Model 4:						
SD Effect(Post SD vs. Pre SD)	1.7	**	2.3	**	1.5	*
Higher social support	1.5	**	1.5	#	1.4	*

# *p* < 0.1,* *p* < 0.05, ** *p* < 0.01, *** *p* < 0.0001.

**Table 6:** Effect of spouse death on depression: stratification analysis.

		Total		Male		Female	
		OR	*p*	OR	*p*	OR	*p*
Stratified by time since SD							
<6 months	Post SD vs. Pre SD	6.0	***	Infinite	--	4.00	**
6–12 months	Post SD vs. Pre SD	0.9	NS	1.00	NS	0.88	NS
12–24 months	Post SD vs. Pre SD	1.7	*	2.38	*	1.41	NS
≥24 months	Post SD vs. Pre SD	1.3	NS	1.89	NS	1.18	NS
Stratified by living arrangement after SD							
Live alone after SD	Post SD vs. Pre SD	2.3	**	3.0	#	2.0	#
Live with family members after SD	Post SD vs. Pre SD	1.5	**	2.3	**	1.3	NS
Stratified by spouse’s heath status before SD(N=548)							
Spouse’s health status: Good	Post SD vs. Pre SD	2.0	**	2.3	*	1.8	*
Spouse’s health status: Not good	Post SD vs. Pre SD	1.3	NS	1.9	NS	1.2	NS

# *p* < 0.1,* *p* < 0.05, ** *p* < 0.01, *** *p* < 0.0001.

## Data Availability

The datasets generated during the current study are not publicly available, but data are however available from the applicants upon reasonable request and with permission of the Ministry of Health and Welfare in Taiwan.
